# A Novel Hyaluronidase Produced by *Bacillus* sp. A50

**DOI:** 10.1371/journal.pone.0094156

**Published:** 2014-04-15

**Authors:** Xueping Guo, Yanli Shi, Juzheng Sheng, Fengshan Wang

**Affiliations:** 1 Key Laboratory of Chemical Biology of Natural Products (Ministry of Education), Institute of Biochemical and Biotechnological Drug, School of Pharmaceutical Sciences, Shandong University, Jinan, China; 2 National Glycoengineering Research Center, Shandong University, Jinan, China; 3 Bloomage Freda Biopharm Co., Ltd., Jinan, China; Universidad de Granada, Spain

## Abstract

Hyaluronidases are a family of enzymes that degrade hyaluronic acid (hyaluronan, HA) and widely used in many fields. A hyaluronidase producing bacteria strain was screened from the air. 16S ribosomal DNA (16S rDNA) analysis indicated that the strain belonged to the genus *Bacillus*, and the strain was named as *Bacillus* sp. A50. This is the first report of a hyaluronidase from *Bacillus*, which yields unsaturated oligosaccharides as product like other microbial hyaluronate lyases. Under optimized conditions, the yield of hyaluronidase from *Bacillus* sp. A50 could reach up to 1.5×10^4 ^U/mL, suggesting that strain A50 is a good producer of hyaluronidase. The hyaluronidase (HAase-*B*) was isolated and purified from the bacterial culture, with a specific activity of 1.02×10^6^ U/mg protein and a yield of 25.38%. The optimal temperature and pH of HAase-*B* were 44°C and pH 6.5, respectively. It was stable at pH 5–6 and at a temperature lower than 45°C. The enzymatic activity could be enhanced by Ca^2+^, Mg^2+^, or Ni^2+^, and inhibited by Zn^2+^, Cu^2+^, EDTA, ethylene glycol tetraacetic acid (EGTA), deferoxamine mesylate salt (DFO), triton X-100, Tween 80, or SDS at different levels. Kinetic measurements of HAase-*B* towards HA gave a Michaelis constant (*K*_m_) of 0.02 mg/mL, and a maximum velocity (*V*_max_) of 0.27 *A*_232_/min. HAase-*B* also showed activity towards chondroitin sulfate A (CSA) with the kinetic parameters, *K*_m_ and *V*_max_, 12.30 mg/mL and 0.20 *A*_232_/min respectively. Meanwhile, according to the sequences of genomic DNA and HAase-*B*’s part peptides, a 3,324-bp gene encoding HAase-*B* was obtained.

## Introduction

Hyaluronidases are a family of enzymes that degrade hyaluronic acid (hyaluronan, HA). The hyaluronidases were classified into three different groups according to their catalytic mechanisms in 1971 by Karl Meyer [1]. The first group is the testicular-type hyaluronidases (EC 3.2.1.35), which degrade HA by cleavage of the β-1,4-glycosidic bond to produce tetrasaccharides as the final products and are found in mammalian spermatozoa, lysosomes and the venoms of various insects and snakes. The second group is hyaluronate-3-glycanohydrolases (EC 3.2.1.36) from leeches and some hookworms, which also degrade HA to tetrasaccharides as the end products, but cleave the β-1,3-glycosidic bond. The last group is microbial hyaluronidases (hyaluronate lyase, EC 4.2.2.1), which degrade HA by eliminating across the β-1,4 linkage and the end products are unsaturated oligosaccharides [2]–[4]. At present, most of hyaluronidases on the market are extracted from animal tissues.

The degradation of HA by hyaluronidases can increase the membrane permeability [5], so hyaluronidases are important drug diffusion agents. Hyaluronidases can accelerate the passage of antibiotics from the circulation into the synovial fluid. They can also improve the systemic bioavailability of proteins [6].

Hyaluronidases, especially bovine testicular hyaluronidase (BTH) preparations, are widely used in many fields like orthopaedics, ophthalmology (vitrectomy), internal medicine, and fertilization [7], [8]. Especially in the course of oncological therapies, hyaluronidase is the only highly effective antidote to the extravasation of the antineoplastic agents like vinblastine, as it reduces the extent of tissue damage and prevents local necrosis by improving the absorption of the extravasates [9], [10]. Furthermore, Zahalka discovered that treatment with hyaluronidase blocks the invasion of tumor cells in an animal model of T cell lymphoma [11].

Another use of hyaluronidases is to degrade HA for producing low molecular weight HA (LMWHA) and HA oligosaccharides. LMWHA and HA oligosaccharides can also be produced by chemical degradation, but chemical degradation will cause the structure changes of HA besides of the cleavage of glycosidic bonds [12]–[15]. So, enzymatic degradation of HA is considered to be highly effective and environmentally friendly.

However, hyaluronidases from animals are limited, but from microbes are unlimited. At present, the microbial hyaluronidases are reported to be obtained from many microorganisms of various genera, including strains of *Clostridium*, *Micrococcus*, *Streptococcus*, and *Streptomyces* [16]–[18]. But, the productivities of microbial hyaluronidases are far from the requirements of industry, even the highest yield reported by Mahesh was 591 U/mL [18], [19]. Due to the medical, physiological, biological and commercial importance of hyaluronidases, it is necessary to screen and isolate a new and more promising strain with higher yield of hyaluronidase. This study reports a novel hyaluronidase produced by *Bacillus* sp. A50.

## Materials and Methods

### Materials

Tryptone and yeast extract were obtained from OXOID Ltd. (Hampshire, England). HA (MW 1×10^6^ Da) was obtained from Bloomage Freda Biopharm Co., Ltd. (Shandong, China). Bovine serum albumin (BSA), chondroitin sulfate A (CSA), dermatan sulfate, deferoxamine mesylate salt (DFO) and hydrolyzed gelatin were purchased from Sigma (USA). Chondroitin sulfate C (CSC) and chondroitin sulfate D (CSD) were purchased from ChromaDex (USA) and Ruibio (German), respectively. Heparan sulphate was purchased from Iduron (UK). Standard hyaluronidase was provided by National Institutes for Food and Drug Control (Beijing, China). All other chemicals were purchased from Sinopharm Chemical Reagent Co., Ltd. (China). Green PCR Master Mix (2×), protein molecular weight standards and DNA marker were purchased from Fermentas (USA). The 16S rDNA universal primers were synthesized by BGI Inc. (Shenzhen, China). Bacteria genomic DNA extraction kit was purchased from Tiandz, Inc. (Beijing, China).

### Hyaluronidase Activity Assay

Hyaluronidase activity was assayed by measuring the reduction of the turbidity resulting from complexes formed between long chains of HA and BSA according to the method described in USP32-NF27 with some suitable modifications. Briefly, 1.0 mg/mL of sodium hyaluronate (previously dried in a vacuum oven with phosphorus pentoxide for 48 h) stock solution was diluted before use with an equal volume of 0.02 M phosphate buffer solution (pH 6.3, dissolve 2.5 g of monobasic sodium phosphate, 1.0 g of anhydrous dibasic sodium phosphate, and 8.2 g of sodium chloride in water to make 1 L). 10 mg/mL of BSA solution (instead of serum stock solution in USP32-NF27) was diluted with 4 volumes of 0.5 M acetate buffer solution (dissolve 14 g of potassium acetate and 20.5 mL of glacial acetic acid in water to make 1 L, and then adjust with 4 M hydrochloric acid to pH 3.1). Standard solution was prepared immediately before use by dissolving standard hyaluronidase in cold hydrolyzed gelatin solution (mix 250 mL of phosphate buffer solution with 250 mL of water and dissolve 330 mg of hydrolyzed gelatin in the mixture) to obtain a solution of 10 U/mL. Sample solution was also properly diluted with cold hydrolyzed gelatin solution before use. The standard concentration-response curve preparation and the assay of test solutions were carried out referring to USP32-NF27, except that the sample detection reaction temperature was changed from 37°C to 42°C.

### Screening of Hyaluronidase Producing Strains

The screening of hyaluronidase producing strains was carried out according to the following procedure. Hyaluronidase producing microorganisms were isolated on plates with selective medium (HA agar medium). The solid medium consisted of 10 g/L peptone, 10 g/L yeast extract, 2 g/L K_2_HPO_4_·3H_2_O, 1 g/L MgSO_4_·7H_2_O, 1 g/L HA, 10 g/L BSA, 20 g/L agar (pH 7.0). The HA agar plates without lids were placed in selected areas in a HA manufacturing workshop. After exposure to the air for 30 min, the plates were covered and incubated at 37°C for 24 h. The bacterial colonies appeared on the plates were transferred to other HA agar plates for backups. Then the plates were soaked in 2 M acetic acid for 10 min [20]. Transparent zones appeared around those colonies which could degrade HA, while the undegraded HA in the agar plate conjugated with BSA to form a white precipitate. The ratio of the diameter of transparent zone to that of the colony was considered as an index for evaluating the productivity of the enzyme by a colony through visual inspection.

The initially screened strains were cultured at 37°C, with a shaking speed of 150 rpm for 18 h in a 50 mL liquid medium composed of 10 g/L tryptone, 10 g/L yeast extract, 2 g/L K_2_HPO_4_·3H_2_O, 1 g/L MgSO_4_·7H_2_O, and 5 g/L glucose (pH 7.0). The strain with the highest hyaluronidase activity was obtained by determining the enzyme activities of each fermentation broth.

Gram staining was carried out at different growth phases of the selected strain in order to observe the morphology of its thallus and spores under an optical microscope.

### PCR Amplification of 16S Rdna and the Sequence Analysis

The selected strain was cultured in LB liquid medium overnight at 35°C. Genomic DNA of the strain was extracted from cells by using Column Bacterial DNAout (Tiandz, China) according to the manufacturer′s instruction. 16S rDNA was amplified from the genomic DNA using certain bacterial universal primers, 27F (5′-AGAGTTTGATCCTGGCTCAG-3′) and 1492R (5′-GGTTACCTTGTTACGACTT-3′) (14), in a 50 µL amplifying system: 1 µL of genomic DNA template, 2 µL of primers 27F and 1492R for each, 25 µL of Green PCR Master Mix (2×) and 20 µL of dH_2_O. The amplification conditions were: initially denaturing at 97°C for 2 min; denaturing at 97°C for 1 min, annealing at 55°C for 1 min, extension at 72°C for 2 min, with 30 cycles; and an additional extension at 72°C for 10 min. The PCR product was identified by DNA agarose gel electrophoresis and then sequenced by BGI Inc. (Shenzhen, China). The obtained 16S rDNA sequence of the selected strain was compared with those deposited in GenBank sequence database using the BLAST program (http://blast.ncbi.nlm.nih.gov/Blast.cgi).

### Purification of the Hyaluronidase Produced by the Selected Strain

The selected strain was cultured in the liquid medium as described above for 8 h to reach the highest hyaluronidase activity. Then, the culture was centrifuged at 15,000×*g*, 4°C for 10 min. Ammonium sulfate powder was added slowly to the supernatant which was meanwhile stirred gently until the concentration of ammonium sulfate reached to 20 g in each 100 mL of the supernatant. The mixture was then gently stirred for an additional 30 min and centrifuged at 15,000× *g*, 4°C for 10 min. The supernatant was decanted and precipitated by addition of another 10 g of ammonium sulfate powder in each 100 mL of the solution. After 30 min standing, the precipitate was collected by centrifugation at 15,000×*g*, 4°C for 10 min, resuspended in 50 mM sodium phosphate buffer solution (pH 6.0) and dialyzed against the same buffer solution. The enzyme solution was purified by anion-exchange chromatography on a 5×30 cm column filled with 50 mL of DEAE Sepharose Fast Flow (GE Healthcare, USA) which had been pre-equilibrated with 50 mM phosphate buffer (pH 6.0). Proteins were eluted with a NaCl linear gradient at a flow rate of 1 mL/min. The fractions with hyaluronidase activity were further purified by gel filtration on a Superdex 200 column, which was eluted with 50 mM phosphate buffer (pH 6.0). Fractions around the hyaluronidase activity peak point were pooled and collected. The purity and molecular weight of hyaluronidase were determined with SDS-PAGE [21].

### Characterization of the Hyaluronidase

#### Optimal pH and temperature

Suitable amounts of the enzyme were used for examining the relative activity under various conditions. To determine the optimal pH, the hyaluronidase activity was measured at 42°C in the range of pH 2.0–12.0 using sodium citrate-Na_2_HPO_4_ buffer (pH 2.0–5.0), Na_2_HPO_4_-NaH_2_PO_4_ buffer (pH 6.0–8.0), Na_2_CO_3_-NaHCO_3_ buffer (pH 9.0–10.0) and Na_2_HPO_4_-NaOH buffer (pH 11.0–12.0). In another series of experiments, temperature settings of 25°C-60°C were used to study the temperature effect at pH 6.0.

#### Thermostability and acidic stability

The thermostability of the hyaluronidase was determined by measuring the residual activity of the enzyme after incubated at 40°C, 45°C, 50°C or 55°C for different time. The pH stability of the hyaluronidase was determined by measuring the residual activity of the enzyme after the enzyme was incubated in the broad pH buffer from pH 4.0 to 12.0 at 25°C for different time. Then the activities of the pre-treated hyaluronidases were measured.

#### Effects of metal ions, metal ion chelators and surfactants on the activity of the hyaluronidase

The effects of several metal ions (Ca^2+^, Ba^2+^, Ni^2+^, Zn^2+^, Co^2+^, Cu^2+^, and Mg^2+^) and metal ion chelators (EDTA, EGTA and DFO) on the hyaluronidase activity were investigated at three different concentrations (10, 50 and 100 mM), and the effects of several surfactants (SDS, Triton X-100, and Tween-80) were studied at four different concentrations (0.5, 1, 1.5 and 2%, w/v). Each of the metal ions, metal ion chelators or surfactants was pre-incubated with the hyaluronidase at room temperature for 30 min, and then the enzyme activity was measured. The enzyme solution without any metal ions, metal ion chelators or surfactants was taken as the control.

#### Substrate specificity of the hyaluronidase

The activity of the hyaluronidase towards different substrates was determined using the method of Oettl [22]. Initially, 0.1 mL of properly diluted enzyme was incubated with 9.9 mL of 10 mg/mL substrate solution in 5 mM phosphate buffer (pH 6.0) at 42°C for 30 min. The hyaluronidase digestions were stopped by heat inactivating the enzyme at 100°C for 2 min and the solution with early inactivated enzyme (inactivated immediately after enzyme and substrate solutions mix) served as the control. The hyaluronidase activity was ascertained by measuring the absorbance of the generated products at the wavelength of 232 nm. The substrates included HA, CSA, CSC, CSD, dermatan sulfate, sodium heparin, heparan sulphate, sodium alginate, chitosan, chitin, sodium carboxymethyl cellulose, dextran 20, carboxymethyl dextran, xanthan gum, gellan gum, soluble starch, carrageenan, konjac glucomannan, synanthrin, gumacabic powder, glycogen, and pectin.

The kinetic parameters, Michaelis constant (*K*_m_) and maximal velocity (*V*_max_) of the hyaluronidase toward HA and CSA, were measured based on the initial reaction rates determined with the concentration ranges of 0.02–0.16 mg/mL of HA and 10–50 mg/mL of CSA at 42°C.

### Determination of the Hyaluronidase Gene

The *N*-terminal amino-acid residue sequence of hyaluronidase was determined by the conventional Edman degradation method using an ABI 491A sequencer, and the peptide fragment fingerprint was analyzed using ESI Q-TOF2 by the Nation Center of Biomedical Analysis (Beijing, China). The genome sequence of the selected strain was sequenced by BGI Inc. (Shenzhen, China). By comparing the sequences of the peptides and genome, the DNA fragment encoding the hyaluronidase was determined.

### Bioinformatics Analysis

The predictions of the molecular weight (MW), isoelectric point (pI) and signal peptide of the hyaluronidase were performed using the tools ExPaSy (http://www.expasy.org) and SignalP 3.0 server (http://www.cbs.dtu.dk/services/SignalP/), respectively. The Blastp program and conserved domain architecture retrieval tool were used to search for similar proteins and conserved domains, respectively (http://www.ncbi.nlm.nih.gov/blast/Blast.cgi).

## Results

### Screening of Strains Producing High-level Hyaluronidase

**Figure 1:**
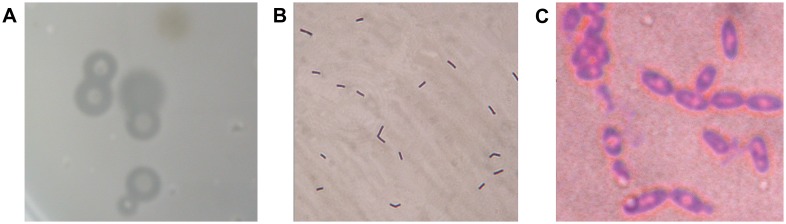
Morphological images of Strain A50. (A) Transparent zones appeared around some colonies on a HA agar plate after soaked in 2 M acetic acid; (B) Gram staining of strain A50 after being cultured for 12 h, showing rod shaped and gram-positive bacteria under the microscope (Magnification is 15×100); (C) Gram staining of strain A50 after being cultured for 24 h, showing spores under the microscope (Magnification is 15×100).

At the first screening, eighteen strains were obtained according to the ratio of the diameter of transparent zone to that of the colony. One plate with the transparent zone was showed in Fig. 1A.

At the second screening, a colony which produced the largest ratio of the diameter of transparent zone to that of the colony, named as A50, was selected for further study. The strain A50 was a rod like, gram-positive and spore-forming bacterium (Fig. 1B and Fig. 1C). Its colonies were circular, pale-yellow with an orderly rim. After inoculating strain A50 into a 250 mL conical flask containing 50 mL of optimized medium and shaking at 37°C for 18 h, the hyaluronidase activity in the culture fluid reached 1.5×10^4^ U/mL, which was the highest level in the existing reports. It solved the problem of the low productivity of microbial hyaluronidases.

### Identification of Strain A50

The 16S rDNA of strain A50 was sequenced and submitted to GenBank with accession number of KC522837. Homologous analysis based on the known 16S rDNA sequences in NCBI revealed that the strain A50 had 99% identity with many *Bacillus* strains. Therefore, the strain A50 was one kind of *Bacillus*, and we named it *Bacillus* sp. A50.

### Purification of the Hyaluronidase Produced by *Bacillus* Sp. A50

**Figure 2:**
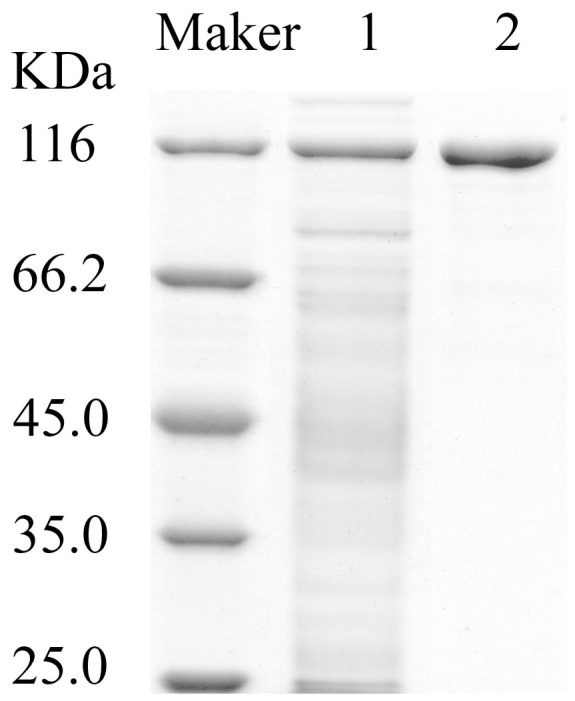
SDS-PAGE of hyaluronidase (HAase-*B*) after Superdex 200 column chromatography. Marker: Unstained Protein Molecular Weight Marker; Lane 1: supernatant of culture fluid; Lane 2: purified hyaluronidase (HAase-*B*).

After salting out, ion-exchange and gel filtration chromatography, the purity of hyaluronidase produced by *Bacillus* sp. A50 was 21-fold of the starting culture medium, with a specific activity of 1.0214×10^6^ U/mg protein and a final yield of 25.38% (Table 1). On a DEAE Sepharose Fast Flow column, the protein was eluted with 0–0.5 M NaCl. The fraction of each peak was collected to analyze the hyaluronidase activity. It was found that only one of the protein peaks had hyaluronidase activity. After further purification on a Superdex 200 column, a single protein band on the SDS-PAGE gel was obtained with the MW of approximately 120 kDa, which was named as HAase-*B* (Fig. 2).

**Table 1:** **Purification of the hyaluronidase produced by** ***Bacillus*** **sp. A50.**

Purification step	Total volume (mL)	Total Protein (mg)	Total activity (×10^6^, U)	Specific activity (×10^4^, U/mg)	Purification fold	Yield (%)
Culture supernatant	1000	309	15.01	4.86	1	100
Ammonium sulphate precipitation	100	43.2	9.66	22.36	4.60	64.36
Ion exchange	48	7.56	6.58	87.04	17.91	43.84
Gel filtration	16	3.73	3.81	102.14	21.02	25.38

### Effects of Temperature and pH on HAase-*B*

**Figure 3:**
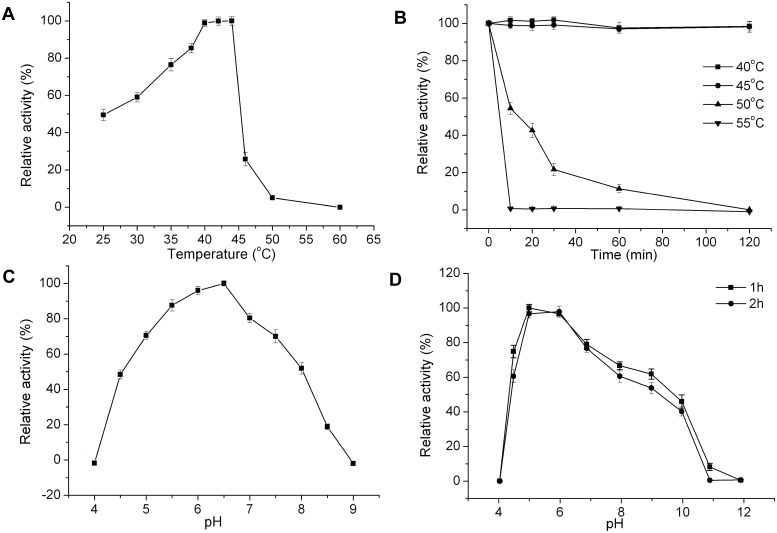
Effects of temperature and pH on the activity and stability of HAase-*B*. (A) Effect of temperature on HAase-*B* activity. The activity of HAase-*B* was measured at different temperatures. (B) Thermal stability of HAase-*B*. The residual activity was assayed in phosphate buffer (pH 6.0) at 42°C. (C) Effect of pH on HAase-*B* activity. The activity was measured at 42°C in a broad pH range buffer as described previously. (D) pH stability of HAase-*B*. Residual activities after incubation at various pH buffer solutions for 1 h or 2 h were assayed at pH 6.0 and 42°C. The graph shows data from triplicate experiments (mean ± SD).

With HA as substrate, HAase-*B* displayed an optimal temperature of 44°C (Fig. 3A). The activity of HAase-*B* remained high and relatively stable between 40°C and 44°C, but only 25% of maximum activity at 46°C. When the temperature reached 60°C or higher, HAase-*B* would lose its activity completely. After incubation at 40°C or 45°C for 120 min, HAase-*B* showed little change with its activity, but its stability was very low at 55°C. The activity reduced by 99% after incubation at 55°C for 10 min (Fig. 3B). The optimum pH for HAase-*B* was 6.5, and there was no detectable activity at pH 4.0 and pH 9.0 (Fig. 3C). HAase-*B* was most stable at pH 5.0–6.0 and retained more than 90% activity after incubation at this pH range for 2 h (Fig. 3D).

### Effects of Metal Ions, Metal Ion Chelators and Surfactants on HAase-*B*

**Figure 4:**
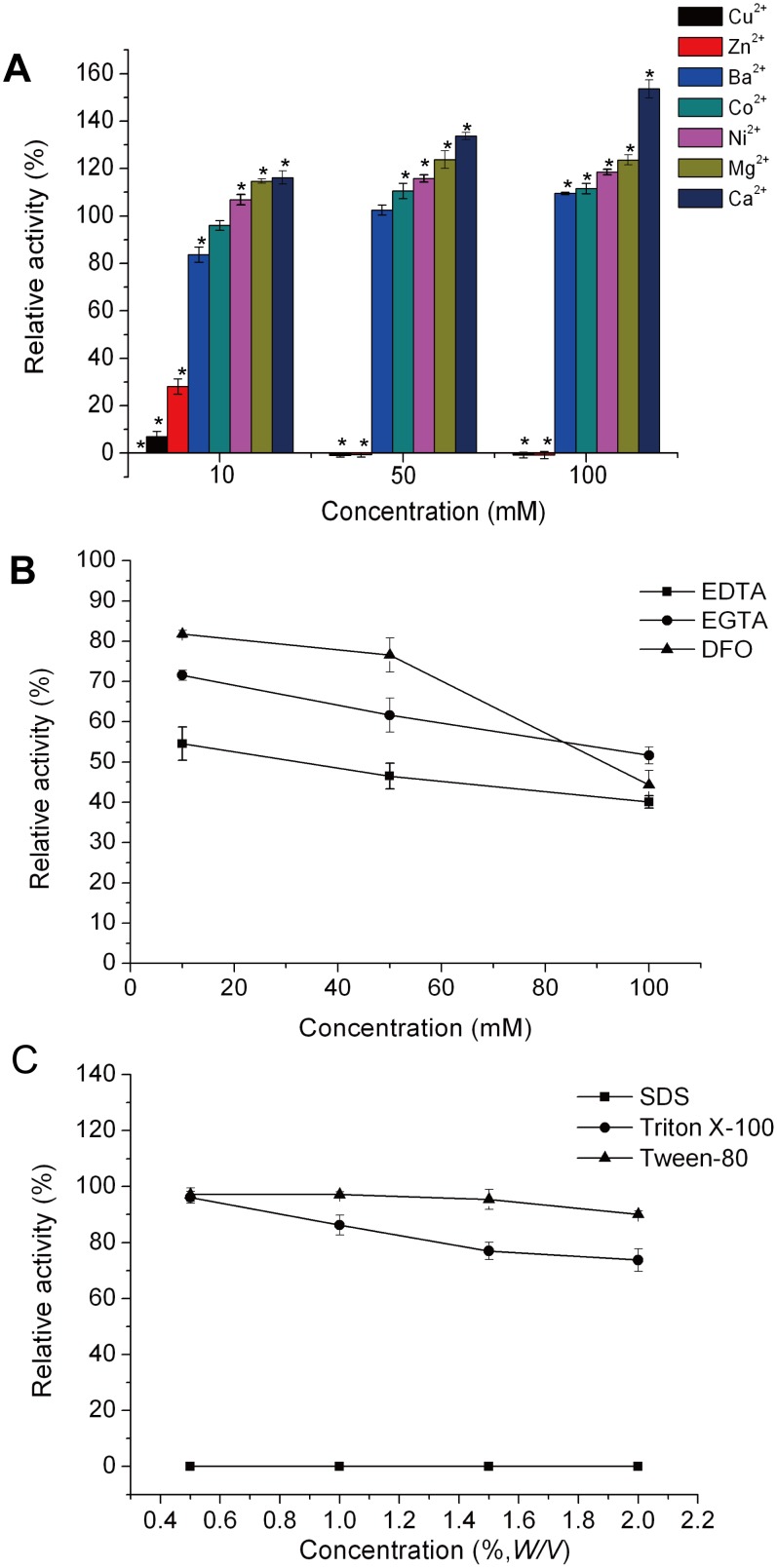
Effects of metal ions, metal ion chelators and surfactants on the activity of HAase-*B*. (A) The influence of various metal ions on HAase-*B* (*p<0.05. The activity of HAase-*B* without any metal ion was taken as control). (B) The influence of metal ion chelators on HAase-*B*. (C) The influence of several surfactants on HAase-*B*. The graph shows data from triplicate experiments (mean ± SD).

The influence of various metal ions (Ca^2+^, Ba^2+^, Ni^2+^, Zn^2+^, Co^2+^, Cu^2+^, and Mg^2+^), metal ion chelators (EDTA, EGTA, and DFO) and surfactants (SDS, Triton X-100, and Tween 80) on HAase-*B* was investigated at different concentrations under the condition with pH of 6.5 and temperature of 44°C. Most of the investigated metal ions, including Ca^2+^, Mg^2+^, Ni^2+^, Co^2+^ and Ba^2+^, had positive effects on the activity of HAase-*B*. HAase-*B* showed the highest activity in 100 mM CaCl_2_ solution, with activity increased to 154%. Zn^2+^ (10 mM) or Cu^2+^ (10 mM) severely inhibited the activity of HAase-*B* by more than 70% and completely inhibited the enzyme activity at the concentrations of 50 mM and 100 mM (Fig. 4A). These effects could be explained by the enzyme conformation modification because of the interaction of metal ions with amino acid residues involved in their active sites. Such interactions can either increase or decrease the enzyme’s catalytic activity [23]. Although the activity of HAase-*B* was decreased by metal ion chelators (EDTA, EGTA and DFO), it was not inhibited completely even when the concentrations of metal ion chelators were raised to 100 mM (Fig. 4B). The experimental results shown in Fig. 4C indicated that non-ionic surfactants (Tween 80 and Triton X-100) moderately inhibit HAase-*B* activity by 3%–30% at different concentrations. However, ionic detergent (SDS) completely inhibited the enzyme activity at any concentrations. The explanation for inhibitory effect of the ionic detergent (SDS) lies in protein tertiary structure alteration to a greater extent due to the electrostatic charge of SDS.

### Substrate Specificity of HAase-*B*

Unlike the most specific lyase prepared from *Streptomyces hyalurolyticus* which degrades only hyaluronan and not the other glycosaminoglycans, HAase-*B* was found to degrade HA, CSA, CSC and CSD, and release unsaturated products which showed a strong absorption peak at 232 nm. When using other polysaccharides as substrate, it did not show any activity (Table 2).

**Table 2:** **Substrate specificity of HAase-** ***B.***

Substrate	Relative activitya (%)
Sodium hyaluronate	100
Chondroitin sulfate A	39.0
Chondroitin sulfate C	13.5
Chondroitin sulfate D	37.1
Dermatan sulfate	0
Heparin sodium	0
Heparan sulfate	0
Sodium alginate	0
Chitosan	0
Chitin	0
Sodium carboxymethyl cellulose	0
Dextran 20	0
Carboxymethyl dextran	0
Xanthan gum	0
Gellan gum	0
Soluble starch	0
Carrageenan	0
Konjac glucomannan	0
Synanthrin	0
Gumacabic powder	0
Glycogen	0
Pectin	0

a The activities of HAase-*B* on various substrates were measured as described in materials and methods. The activity of HAase-*B* on sodium hyaluronate was taken as 100%. The data represent the mean of three experimental repeats with SD≤5%.

**Figure 5:**
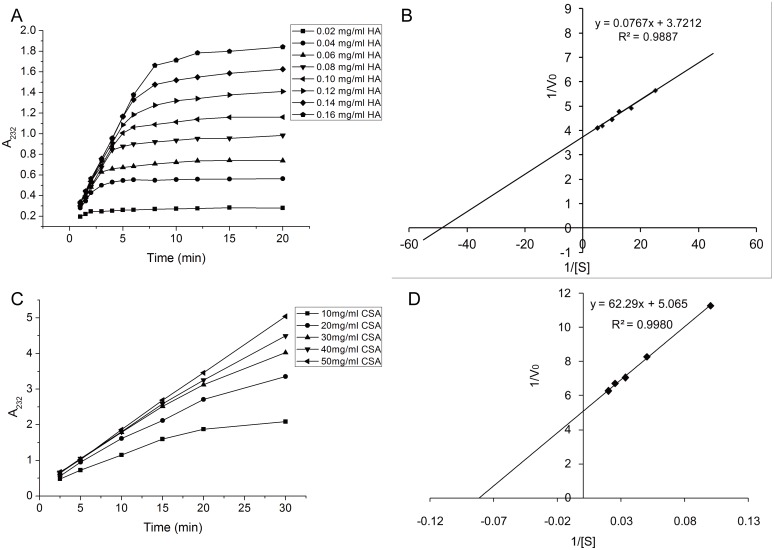
Enzymatic reaction kinetics of HAase-*B* towards HA and CSA. (A) Time dependent kinetics of HAase-*B* in digesting HA substrate of various concentrations. (B) The Lineweaver–Burk double reciprocal plot showing 1/V_0_ versus 1/[S] (R^2^ = 0.9950), V_0_ represents initial reaction rate, and [S] represents HA substrate concentration. (C) Time dependent kinetics of HAase-*B* in digesting CSA substrate of various concentrations. (D) The Lineweaver–Burk double reciprocal plot showing 1/V_0_ versus 1/[S] (R^2^ = 0.9980), herein, V_0_ represents initial reaction rate, and [S] represents CSA substrate concentration.

The substrate kinetic parameters of HAase-*B*, *K*_m_ and *V*_max_, were estimated from a series of steady state initial reaction rates *V*_0_ (*A*_232_/min) measured at various substrate concentrations (Fig. 5A) as described in the preceding section and calculated according to the following formula
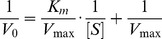



According to the formula, the experimental results were plotted according to the Lineweaver–Burk double reciprocal models (Fig. 5B), which rearranged the Michaelis–Menten equation and resulted in kinetic values with HA as substrate of 0.02 mg/mL and 0.27 *A*_232_/min for *K*_m_ and *V*_max_, respectively. The kinetic parameters of HAase-*B* towards CSA were obtained in the same way (Fig. 5C and Fig. 5D).

The *K*_m_ values of HAase-*B* were calculated to be 0.02 mg/mL and 12.30 mg/mL (Table 3) with HA and CSA as substrate respectively.

**Table 3:** **Kinetic parameters of HAase-** ***B*** **towards HA and CSA.**

Substrate	HA	CSA
*K*m (mg/mL)	0.02	12.30
*V*max (*A*232/min)	0.27	0.20

### Sequence Analysis and Gene Sequencing of HAase-*B*

The sequences of *N*-terminal amino acids and seven peptide fragments of HAase-*B* were determined as follows: NESTLLLNTS (*N*-terminal), NDSAVQAVQDVK, LLKALLAPATAFAPK, TPANADSLR, YSVLKEDTYLDYFK, KSTAVLTVEYGSLGPR, QSAGPLTVYAGK, NESTLLLPWLCLYASGDK. Peptide mass fingerprint attested this hyaluronidase was a new protein.

The incomplete genome sequence of *Bacillus* sp. A50 composed of 63 scaffolds was acquired by Illumina Genome Analyser Sequencing technology. By comparing the sequences of the peptides and scaffolds, a DNA fragment of 3,324 bp encoding 1,106 amino acid residues was determined to be the gene encoding HAase-*B*. This DNA fragment was located on scaffold 21 which contains 89,637 bp. After comparing scaffold 21 with GenBank database of NCBI, we found that part sequences of scaffold 21 and those of complete genome of *Bacillus* had similarities of about 80%. This can demonstrate that scaffold 21 is part of the bacterial genome. Hence, the gene encoding HAase-*B* is part of genome and not encoded on a plasmid. The DNA sequence of HAase-*B* obtained in our work was submitted to GenBank with KC 522838 as its accession number. The MW and pI of HAase-*B* were predicted to be 123,242 Da and 5.05, respectively.

The deduced amino acid sequence of HAase-*B* had relatively high identity to those of other members of glycosaminoglycan (GAG) lyase family. The alignment result showed the highest similarity with HAase-*B* sequence occurred in a precursor enzyme of Xanthan gum lyase XalB from *Paenibacillus*, up to 46% similarity, and 31% to 46% similarities with all the other proteins of GAG lyase family. Compared with the conserved regions of all the GAG lyases above, the active sites of HAase-*B* might be N513, H563 and Y572. By alignment of the deduced *Bacillus* sp. A50 HAase-*B* amino acid sequence with the GAG lyase family enzymes, we also found the substrate binding sites of HAase-*B* might be R406, T409, K413, N453, W455, H456, G460, N463, S467, N513, K519, H563, Y572, V575, E578, D582, R626, A627, R630, M741, and N742.

## Discussion

HA fragments, which perform different biological functions according to its molar mass such as angiogenesis, induction of endothelial cell differentiation, stimulation of fibroblast proliferation and synthesis of collagen [24]–[26], have attracted more and more attention. Due to the non-specificity of acid degradation, besides scission of glycosidic bonds, lots of side reactions would occur and lead to structural changes of disaccharide units. Thus the method to produce LMWHA and HA oligosaccharides by hyaluronidase is the best one as a result of its high efficiency and mildness of reaction conditions.

In this study, screening of hyaluronidase producing organism was carried out in a HA manufacturing workshop. The relatively high content of HA in the air might be the inducing factor of generating organisms that produce and secrete high levels of hyaluronidase. The high activity, mild reaction conditions, and good stability of HAase-*B* suggest that *Bacillus* sp. A50 is a good producer of hyaluronidase and could promote industrial preparation of LMWHA and HA oligosaccharides through enzymatic degradation.

To date, the amino acid sequences of many hyaluronidases from prokaryotes have been determined [18], [27]. The best known and characterized bacterial hyaluronidases are from *Streptococcus pneumoniae* and *Streptococcus agalactiae* [28]–[30]. However, HAase-*B* may be the only hyaluronidase in the existing report which is the major protein product in the culture medium, as reflected in the supernatant gel. It is probably that *Bacillus* sp. A50 was induced by high levels of HA in the environment to produce more hyaluronidase. The nucleotide sequence of hyaluronidase (HAase-*B*) gene from *Bacillus* sp. A50 has been determined, with GenBank accession number of KC 522838, which introduces new information to the data of bacterial hyaluronidases. Among the bacterial hyaluronidases of which the deduced amino acid sequence is known, there is a wide variation in the molecular weights, as represented by *S. pneumonia* (107 kDa), *S. agalactiae* (121 kDa), *Clostridium perfringens* (114 kDa), *Streptococcus aureus* (92 kDa), *Propionibacterium acnes* (82 kDa) and two enzymes from *Streptomyces* sp. (77 and 84 kDa). The molecular weights of other non-sequenced hyaluronidases also vary from 50 to 160 kDa [27], [31], [32]. In this article, the molecular weight of hyaluronidase from *Bacillus* sp. A50 is approximately 123 kDa.

Many bacterial hyaluronidases appear to degrade HA through initial endolytic cuts followed by processive exolytic cleavage of one disaccharide at a time [17], [29], [33], [34]. According to structural properties of HA and these hyaluronidases, it seems that such degradation initiates as an endolytic, random bite process for high molecular weight HA and as HA chains become smaller the processive, exolytic degradation takes over. However, due to the little similarity between the deduced amino acid sequences of hyaluronidase from *Bacillus* sp. A50 and other bacterial hyaluronidases above, further studies are needed to elucidate the degradation mechanism.
